# Characterization of the Early Life Microbiota Development and Predominant *Lactobacillus* Species at Distinct Gut Segments of Low- and Normal-Birth-Weight Piglets

**DOI:** 10.3389/fmicb.2019.00797

**Published:** 2019-04-16

**Authors:** Na Li, Shimeng Huang, Lili Jiang, Zhaolai Dai, Tiantian Li, Dandan Han, Junjun Wang

**Affiliations:** State Key Laboratory of Animal Nutrition, College of Animal Science and Technology, China Agricultural University, Beijing, China

**Keywords:** low birth weight, gut microbiota, *Lactobacillus* species, gut segments, piglet

## Abstract

Microbial exposure during early life plays a pivotal role in modulating the health and intestinal development of the host. Our recent study showed that the low-birth-weight (LBW) piglets harbored a different fecal microbiota compared to normal-birth-weight (NBW) piglets during early life with a lower abundance of the genus *Lactobacillus*. Considering the spatial variations in gut microbiota at distinct gut locations, this study was designed to further investigate the differences in the microbiota composition and predominant *Lactobacillus* species in the ileum and colon between LBW and NBW piglets during early life, including day 7 (D7), day 21 (D21, before weaning), and day 35 (D35, 2 weeks after weaning). Compared with the normal group, LBW piglets harbored a significantly lower proportion of short-chain fatty acids producing microbes, such as Ruminococcaceae and Prevotellaceae in the ileum on D7, *Alistipes* and Lachnospiraceae in the colon on D7, *Blautia* in the colon on D21, and *Ruminiclostridium 9* in the colon on D35. The relative abundance of the phylum Bacteroidetes was also declined in both ileum and colon of LBW piglets on D7. Meanwhile, the levels of total SCFAs on D7, D21, and D35, acetate and valerate on D7 and D21, propionate on D21, and lactate on D21 and D35, were also declined in the colon of LBW piglets. Moreover, functional alterations in the gut microbiota of LBW piglets were characterized by differentially abundant microbial genes involved in multiple pathways such as amino acid metabolism, energy metabolism, replication and repair, and metabolism of cofactors and vitamins in the colon. Additionally, lower numbers of *L. salivarius* on D7 and *L. amylovorus* on D21 resided in the colon of LBW piglets compared to those in the normal ones. Collectively, LBW piglets have altered bacterial communities, microbial metabolism and gene functions in the ileum and colon during early life, especially the colonic community. This work will help to develop novel ideas in identifying the reliable biomarkers affecting the gut microbiota development in LBW piglets during early life and facilitate the development of new nutritional interventions.

## Introduction

Increased litter size by genetic selection for high-prolific sows in recent years has been accompanied by an increasing occurrence of piglets born with LBW (Li et al., 2017). LBW pigs refer to those pigs with a birth weight less than 1.1 kg, accounting for 15–25% of the newborn piglets (Wang et al., 2017). LBW piglets are more susceptible to high postnatal morbidity and mortality, postnatal growth restriction (Wu et al., 2006), as well as malfunction in vital organs like the GIT (Li et al., 2017; Wang et al., 2018). Previous studies have revealed a significant alteration of the small intestinal development in LBW fetuses (Wang et al., 2014) as well as LBW piglets at birth (Wang et al., 2008) and during lactation (Wang et al., 2010).

A highly diverse microbial consortium inhabits in the mammalian GIT and plays a vital role for their host (Rooks and Garrett, 2016). Early life colonization of microbes in the gut of the piglet sets the stages for adult microbiota and has lifelong influences on the development of the GIT and immune system (Matamoros et al., 2013; Houghteling and Walker, 2015). On the other hand, the gut microbiota of newborns is extremely unstable due to the fast growth of the GIT and its dysbiosis is closely connected with a higher risk of gut diseases and infections (Houghteling and Walker, 2015).

Several studies have investigated the differences in the early establishment of gut microbiota between LBW and NBW piglets. LBW piglets present a significantly distinct fecal bacterial community structure compared with NBW piglets during suckling and weaning periods (Li et al., 2018). D’Inca et al. (2010, 2011) showed that intrauterine growth restricted (IUGR) pigs with LBW had higher counts of adherent bacteria in ileal and colonic mucosa compared with the normal ones via a traditional colony-counting method. The distribution of gut microbes in pigs exhibits spatial heterogeneity across different intestinal segments, with Firmicutes and Proteobacteria dominating in the small intestine, while Firmicutes and Bacteroidetes dominate in the large intestine (Zhao et al., 2015; Mu et al., 2017). Therefore, it will be necessary to disclose the differences in the bacterial community between LBW and NBW piglets at distinct gut locations by using high-throughput 16S rRNA gene sequencing. In addition, the gut microbiota, particularly the microbiota in the hindgut, can ferment the carbohydrates to produce SCFAs, which are beneficial to the intestinal physiological functions (Koh et al., 2016). Our recent study showed the population of SCFAs-producing bacteria, including *Prevotella* spp. and *Faecalibacterium* spp., were markedly diminished in feces of the LBW piglets (Li et al., 2018), however, whether production of SCFAs in different gut segments of the LBW piglets is distinct from normal pigs requires further investigations.

Historically, *Lactobacillus* species, such as *Lactobacillus amylovorus* (*L. amylovorus*), *Lactobacillus johnsonii* (*L. johnsonii*), *Lactobacillus mucosae* (*L. mucosae*), and *Lactobacillus salivarius* (*L. salivarius*), have been widely considered to be the predominant endogenous probiotics in the GIT of pigs (Leser et al., 2002; Pieper et al., 2006; Houghteling and Walker, 2015). Our previous study has demonstrated that the proportion of the genus *Lactobacillus* was continuously lower in feces of the LBW piglets than that in NBW piglets during the first 35 days (Li et al., 2018). Further studies are needed to figure out the dynamic changes in dominant *Lactobacillus* species along the GIT in LBW and NBW piglets during their early life.

Therefore, the current study was designed to further characterize the differences in the development of microbiota assembly and predominant *Lactobacillus* species at distinct intestinal locations between LBW and NBW piglets during their early life, including the pre- and post-weaning periods. Moreover, dynamic shifts for production of the bacterial metabolites, including SCFAs and lactic acid, in these pigs were also clarified. The findings of this study are supposed to provide novel evidence for altered development of the gut microbiota in LBW piglets and promote the development of new probiotics for newborns with LBW.

## Materials and Methods

### Animals and Sample Collection

A total of 18 litters of full-term piglets (Landrace × Yorkshire) were selected from a commercial pig breeding farm in Mianyang city, Sichuan province, China. Only sows with 12∼14 live-born piglets were included and no cross-fostering was conducted in the present study. A NBW piglet was selected as a pig having a birth weight within 1 standard deviation (SD) of the mean birth weight of the whole litter, whereas a piglet with a birth weight 2 SD below the mean was labeled as a LBW littermate as we described previously (Wang et al., 2014). Average birth weights for all LBW and NBW piglets in the current study were 0.878 ± 0.044 and 1.434 ± 0.034 kg, respectively. Neonatal piglets were able to suckle the sow and drink water *ad libitum*, as well as started to receive a commercial creep feed from day 3 to 5 postpartum. All the piglets were weaned at day 21 and transferred into the nursery pens with free access to solid feed and water. No antibiotics or other drugs were used during the experiment. On day 7 (D7), day 21 (D21, before weaning), and day 35 (D35, 2 weeks after weaning) after birth, piglets (6 LBW and 6 NBW piglets) from randomly selected 6 litters were killed after anesthesia for sample collection. The digesta from the ileum and colon were immediately collected on ice, placed in liquid nitrogen, and then stored at -80°C until analysis.

### DNA Extraction, 16S rRNA Gene Amplification and Sequencing

Total genomic DNA was extracted from 0.5 g of each specimen using the QIAamp^®^ Fast DNA Stool Mini Kit (Qiagen Ltd., Germany) in accordance with the manufacturer’s protocol. The V3-V4 region of the 16S rRNA gene was amplified using universal primers 338F (ACTCCTACGGGAGGCAGCAG) and 806R (GGACTACHVGGGTWTCTAAT) (Ren et al., 2017). The amplified products were detected using 2% agarose gel electrophoresis, purified using AxyPrep DNA Gel Extraction Kit (Axygen Biosciences, United States), and quantified by Qubit 2.0 Fluorometer (Thermo Fisher Scientific, United States). Purified PCR products were pooled into equimolar amounts and sequenced on the Illumina MiSeq platform to generate paired-end reads of 300 bp (Caporaso et al., 2012).

### Analysis of Sequencing Data

Raw paired-end reads were strictly analyzed using QIIME (version 1.9) (Caporaso et al., 2012). In brief, the low-quality sequences with a length of <220 nt or >500 nt, an average quality score of <20, and sequences containing > 3 nitrogenous bases, were removed (Masella et al., 2012). The remaining high-quality sequences were clustered into OTUs at a 97% similarity using UPARSE (version 7.0) (Edgar, 2013) and chimeric sequences were removed using UCHIME (Edgar et al., 2011). Taxonomy assignment of OTUs was conducted with the RDP classifier^1^ (Bacci et al., 2015) against the SILVA 16S rRNA gene database (Release128)^2^ (Pruesse et al., 2007) with a confidence threshold of 0.70.

Alpha-diversity was evaluated by calculating the Shannon diversity index and number of OTUs per sample with the MOTHUR program (version v.1.30.1)^3^ (Schloss et al., 2009). Bar plots and Heat maps were obtained using the “vegan” package in R (version 3.3.1). For beta-diversity analysis, PCoA was performed based on Bray-Curtis and Unweighted Unifrac distances using QIIME (version 1.9). ANOSIM (1,000 Monte Carlo permutations) based on Bray-Curtis and Unweighted Unifrac distances was performed to compare the similarity of microbial community between groups using the “vegan” package of R (version 3.3.1). In addition, the prediction of the microbial gene functions were done using PICRUSt software (version 1.0) against the KEGG database (Langille et al., 2013).

### Determination of the Bacterial Metabolites

Short-chain fatty acids including formate, acetate, propionate, butyrate, isobutyrate, valerate, and isovalerate, as well as lactate in luminal contents were quantified with Ion Chromatograph as previously described (He et al., 2018). In brief, 0.5 g of digesta samples were weighed, dissolved with 8 mL ultrapure water, homogenized, and then centrifuged at 3000 × *g* for 10 min. The supernatant was diluted (1:50), filtered through a 0.22 μm membrane, kept in a 2 mL screw-cap vial, and then subjected for SCFAs analysis with an Ion Chromatography System (DIONEX ICS-3000, Thermo Fisher Scientific, United States).

### Quantification of Predominant *Lactobacillus* Species

Total DNA was extracted from the luminal digesta samples as mentioned above. The primers for the species *L. amylovorus*, *L. johnsonii*, *L. mucosae*, and *L. salivarius*, total *Lactobacillus*, and total bacteria are shown in Supplementary Table S1 as reported previously. The qPCR was conducted with the Roche LightCycler^®^ 96 Real-time PCR system (Roche, Sweden). The reaction mixture (25 μL) contained 1.5 μL forward and 1.5 μL reverse primers, 12.5 μL 2 × TB Green^TM^ Premix Ex Taq^TM^ II (Takara, Japan), 1 μL template DNA, and 8.5 μL ddH_2_O. The reaction protocol consisted of one initial denaturation at 95°C for 10 min, 40 cycles of denaturation at 95°C for 10 s, 60 s at the appropriate annealing temperature (Supplementary Table S1), and extension at 72°C for 10 s. All samples were analyzed in duplicates. Standard curves were generated by constructing standard plasmids containing the 16S rRNA genes as previously described (Han et al., 2012). The copy numbers of each target bacteria were calculated using the corresponding standard curves. Briefly, a series of 10-fold dilution (10^9^ to 10^1^ copies/μL) of plasmid DNA were used to generate their respective standard curves with the logarithm of target copy numbers as the abscissa and the Ct values as the ordinate. The gene copy numbers were calculated using the following equation: [DNA concentration (μg/μL) × 6.0233 × 10^23^ copies/mol]/[DNA size (bp) × 660 × 10^6^].

### Statistical Analysis

Non-parametric Mann–Whitney *U*-test (SPSS 20.0) was used to compare the difference between LBW and NBW piglets as well as between the ileal and colon at each time-point (Ren et al., 2016). *P*-values were adjusted with a FDR (below 5%) as described by Benjamini and Hochberg (1995). The differential bacteria taxa between LBW and NBW piglets were identified using discriminant analysis (LDA) effect size (LEfSe) analysis. Moreover, STAMP software (version 2.1.3) was applied to detect the differentially abundant KEGG pathways between groups using the Wilcoxon rank-sum test with FDR correction (Parks et al., 2014). Only taxa with an average relative abundance greater than 0.01% were considered. The corrected *P*-values below 0.05 were considered as statistically significant.

## Results

### Summary of Sequencing Data and Alpha Diversities Across All Samples

A total of 2,683,338 high-quality sequences were obtained from 72 digesta samples, with an average of 37,268 sequences per sample. Rarefaction curves implied that almost all the bacterial species were captured in luminal contents of all piglets (Supplementary Figure S1). We randomly subsampled per sample to 23,185 sequences for subsequent analysis. These sequences were clustered into 1,250 OTUs based on 97% sequence similarity, and then assigned to 21 phyla, 40 classes, 85 orders, 167 families, and 441 genera.

**Figure 1:**
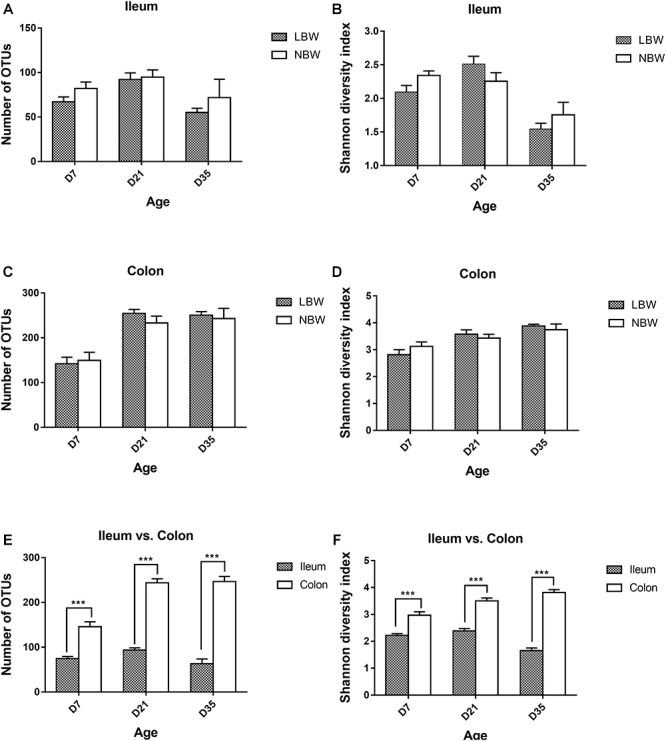
Alpha diversity of the gut bacterial community of LBW and NBW piglets. Number of OTUs **(A)** and shannon diversity index **(B)** in ileal samples of LBW and NBW piglets at each time point. Number of OTUs **(C)** and shannon diversity index **(D)** in colonic samples of LBW and NBW piglets at each time point. Number of OTUs **(E)** and Shannon diversity index **(F)** in the colon and ileum of all piglets at each time point. Data are shown as mean ± SEM. ^∗∗∗^*P* < 0.001. *n* = 6 per group. LBW, low birth weight; NBW, normal birth weight.

Alpha diversity parameters, including Numbers of OTUs and Shannon diversity index, were substantially increased in the colon compared with the ileum at each time-point (Figure 1E,F). However, birth weight had no significant influence on the bacterial richness and diversity of ileum and colon at any time-point (Figure 1A–D).

### Spatial Variations in the Ileal and Colonic Microbiota

**Figure 2:**
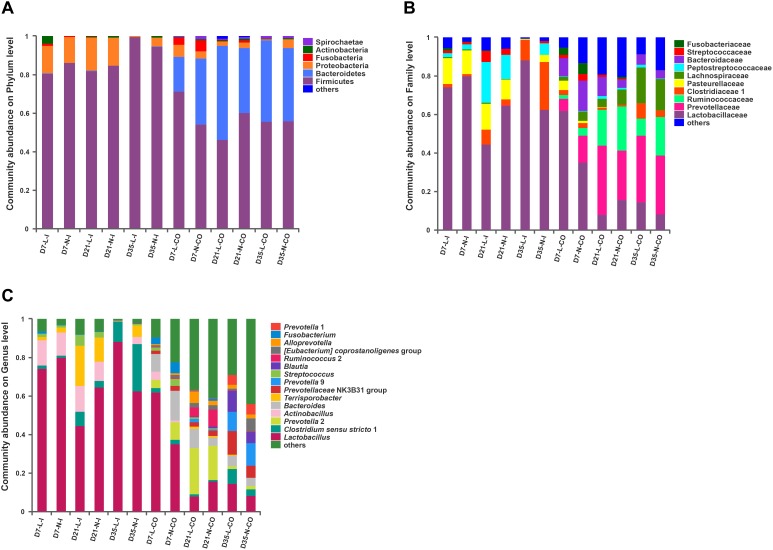
Gut microbiota composition of LBW and NBW piglets. Abundant phyla **(A)**, families **(B)**, and genera **(C)** in the gut microbiota of LBW and NBW piglets. Only families and genera with average relative abundance greater than 5% were shown. Data are shown as means. *n* = 6 per group. L, low birth weight; N, normal birth weight; I, ileum; CO, colon.

**Figure 3:**
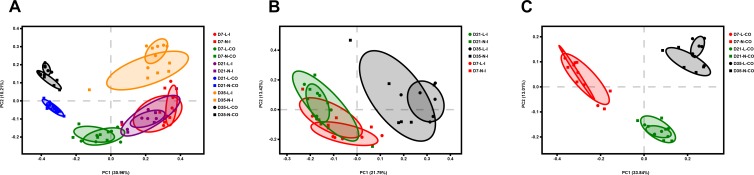
Principal coordinate analysis (PCoA) of all samples **(A)**, ileal samples **(B)**, and colonic samples **(C)** of LBW and NBW piglets, based on Unweighted UniFrac distances. *n* = 6 per group. L, low birth weight; N, normal birth weight; I, ileum; CO, colon.

The overall bacterial composition of the ileum was significantly different from the colon throughout this experiment, irrespective of birth weight (Figure 2). A PCoA plot based upon Bray-Curtis distances (Supplementary Figure S2A) and Unweighted Unifrac distances (Figure 3A) further confirmed that ileal samples were clearly separated from colonic samples at all time-points. An ANOSIM of these distances also showed that the gut microbiota structure of piglets was strongly affected by gut segments (Supplementary Table S2, *P* < 0.01). At the phylum level (Figure 2A), Firmicutes and Proteobacteria were predominant in the ileum, while Firmicutes and Bacteriodetes were dominant in the colon. At the family level (Figure 2B), Lactobacillaceae, Clostridiaceae 1, Pasteurellaceae, Peptostreptococcaceae, and Streptococcaceae were the main bacterial families in ileal samples. In contrast, Prevotellaceae, Ruminococcaceae, Bacteriodaceae, Fusobacteriaceae, as well as Lactobacillaceae were dominant in colonic contents. At the genus level (Figure 2C), ileal microbiota was dominated by *Lactobacillus*, *Actinobacillus*, *Terrisporobacter*, *Streptococcus*, and *Clostridium sensu strico* 1. In the colon, *Lactobacillus* was still dominant but significantly lower than that in the ileum. Meanwhile, the relative proportions of *Bacteroides*, five genera of Prevotellaceae (*Prevotella* 2, *Prevotella* 1, *Prevotella* 9, *Alloprevotella*, *Prevotellaceae* NK3B31 group), *Fusobacterium*, and *Ruminococcus* 2 were greater compared with the ileal microbiota. The relative abundances of the top 20 most abundant genera are presented in Supplementary Figure S3 for visualization.

### Differences in the Ileal and Colonic Microbiota Between LBW and NBW Piglets

Principal coordinates analysis plots based on Bray-Curtis distances (Supplementary Figures S2B,C) and Unweighted Unifrac distances (Figure 3B,C) showed no clear separation of the overall bacterial community structure between LBW and NBW piglets on D7, D21, and D35, which was confirmed by ANOSIM (Supplementary Table S2, *P* > 0.05).

**Figure 4:**
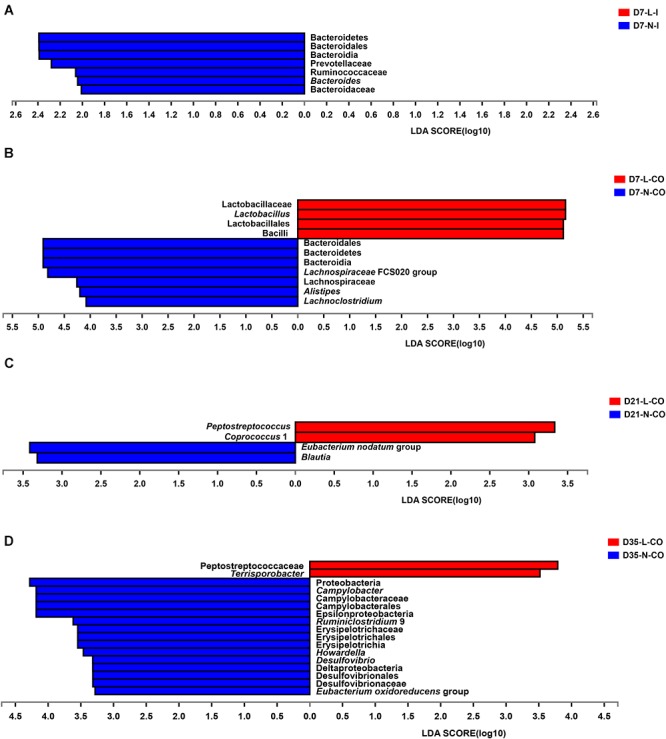
Differentially abundant taxa between LBW and NBW piglets. Histograms of a linear discriminant analysis (LDA) score (threshold: ≥ 2) in ileal samples on D7 **(A)**, colonic samples on D7 **(B)**, colonic samples on D21 **(C)**, and colonic samples on D35 **(D)** are plotted. *n* = 6 per group. L, low birth weight; N, normal birth weight; I, ileum; CO, colon.

Furthermore, we applied the Mann–Whitney *U*-test and LEfSe analysis to identify differentially abundant phyla, families, and genera in the ileal and colonic microbiota between LBW and NBW piglets at each certain age. The results revealed that the influence of LBW on the bacterial community composition were focused on the colonic digesta (Figure 4 and Supplementary Table S3). At the phylum level, the relative abundance of Bacteroidetes was significantly higher in the ileum (0.006 vs. 0.0 51%, *P* < 0.05) and colon (17.480 vs. 33.860%, *P* < 0.05) of LBW piglets than that in NBW piglets on D7. On D35, LBW piglets had significantly lower population of Proteobacteria in the colon (0.311 vs. 4.418%, *P* < 0.01) compared with the normal ones. At the family level, 7-day-old LBW piglets showed dramatically lower relative abundances of Prevotellaceae (0.006 vs. 0.037%, *P* < 0.05), Ruminococcaceae (0.002 vs. 0.022%, *P* < 0.05), and Bacteroidaceae (0.000% vs. 0.010%, *P* < 0.05) in the ileum as well as Lachnospiraceae (1.924% vs. 4.952%, *P* < 0.05) in the colon than NBW piglets. However, the relative abundance of Lactobacillaceae (62.850% vs. 35.720%, *P* < 0.05) was significantly increased in the colon of LBW piglets compared with the normal group. In the colon on D35, LBW piglets presented markedly lower proportions of Campylobacteraceae (0.000% vs. 3.400%, *P* = 0.01), Desulfovibrionaceae (0.028% vs. 0.343%, *P* < 0.05), and Erysipelotrichaceae (0.384% vs. 0.828%, *P* < 0.05) but higher Peptostreptococcaceae (1.627% vs. 0.423%, *P* < 0.05) than NBW piglets. At the genus level, the lower proportions of *Bacteroides* (0.000% vs. 0.010%, *P* < 0.05) in the ileum, *Alistipes* (0.050% vs. 0.235%, *P* < 0.05), *Lachnoclostridium* (0.556% vs. 2.440%, *P* < 0.05), and *Lachnospiraceae* FCS020 group (0.000% vs. 0.039%, *P* < 0.05) were observed in LBW piglets on D7 compared to normal ones. But a higher proportion of *Lactobacillus* (62.850% vs. 35.720%, *P* < 0.05) resided in the colon of LBW piglets. Moreover, the population of *Peptostreptococcus* (0.010% vs. 0.000%, *P* < 0.05) and *Coprococcus* 1 (0.225% vs. 0.024%, *P* < 0.05) on D21, and *Terrisporobacter* (0.669% vs. 0.118%, *P* < 0.05) on D35 were significantly higher in the colon LBW piglets than those in NBW piglets. In the contrary, *Blautia* (0.291% vs. 0.721%, *P* < 0.05) and *Eubacterium nodatum* group (0.116% vs. 0.525%, *P* < 0.05) had obviously lower proportions in the colon of 21-day-old LBW piglets compared with the NBW group. Meanwhile, relative abundances of *Campylobacter* (0.000% vs. 0.340%, *P* < 0.01), *Desulfovibrio* (0.026% vs. 0.336%, *P* < 0.05), *Eubacterium oxidoreducens* group (0.000% vs. 0.021%, *P* < 0.05), *Howardella* (0.006% vs. 0.043%, *P* < 0.01), and *Ruminiclostridium* 9 (0.325% vs. 1.205%, *P* < 0.05) were obviously reduced in the colon of 35-day-old LBW piglets.

### Differences in the Predicted Microbial Gene Functions Between LBW and NBW Piglets

**Figure 5:**
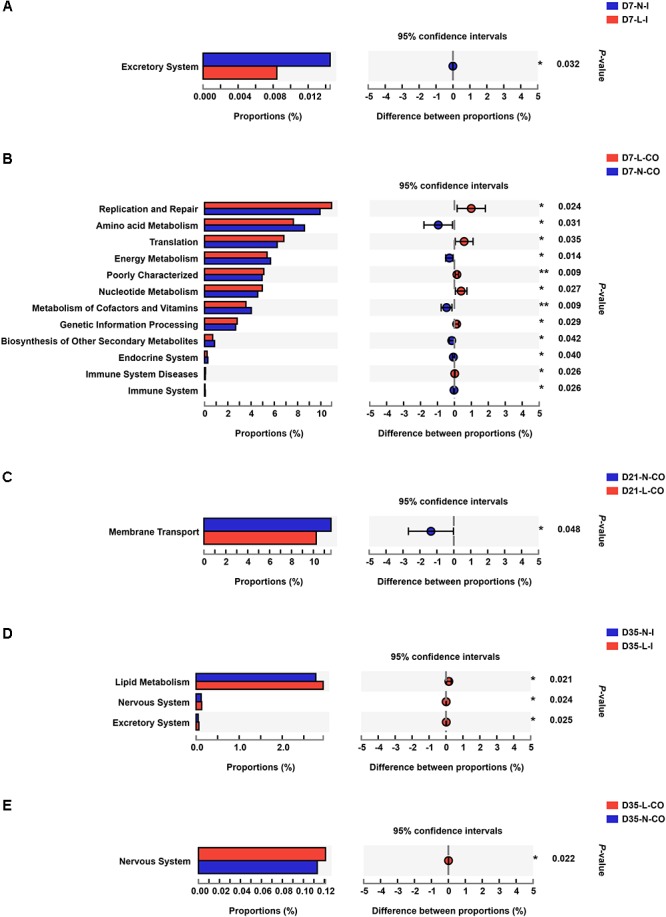
Differentially functional profiles of the gut bacterial community between LBW and NBW piglets. Differential abundant KEGG pathways in ileal samples on D7 **(A)**, colonic samples on D7 **(B)**, colonic samples on D21 **(C)**, ileal samples on D35 **(D)**, and colonic samples on D35 **(E)** are plotted. Data are shown as means. ^∗∗^*P* < 0.01; ^∗^*P* < 0.05. *n* = 6 per group. L, low birth weight; N, normal birth weight; I, ileum; CO, colon.

To figure out the functional differences between microbiome residing in LBW and NBW piglets, we performed functional analysis of microbiota using PICRUST. A total of 41 categories of gene functions were successfully obtained. The most abundant gene functions included membrane transport (10.260∼13.880%), carbohydrate metabolism (10.120∼11.210%), replication and repair (9.458∼11.510%), amino acid metabolism (6.969∼9.649%), translation (6.089∼7.202%), energy metabolism (4.934∼6.151%), poorly characterized (4.802∼5.308%), and nucleotide metabolism (4.309∼5.357%). The results of STAMP demonstrated that the functional capability of LBW and NBW piglets significantly differed at each time-point (Figure 5 and Supplementary Table S4). Compared with NBW piglets, the ileal bacterial community in LBW piglets had higher relative abundances of the genes associated with lipid metabolism (0.325 vs. 1.205%, *P* < 0.05) and nervous system (0.130 vs. 0.119%, *P* < 0.05) on D35. Meanwhile, the colonic community of 35-day-old LBW piglets also have the increased proportion of genes involved in nervous system (0.121 vs. 0.113%, *P* < 0.05). In addition, a total of 12 differentially metabolic pathways were detected in the colonic microbiota between 7-day-old LBW and NBW piglets. The relative abundances of the genes involved in replication and repair (10.920 vs. 9.926%, *P* < 0.05), translation (6.818 vs. 6.244%, *P* < 0.05), poorly characterized (5.097 vs. 4.949%, *P* < 0.05), nucleotide metabolism (4.967 vs. 4.570%, *P* < 0.05), genetic information processing (2.806 vs. 2.674%, *P* < 0.05), and immune system diseases (0.088 vs. 0.069%, *P* < 0.05) were dramatically enriched in the colonic community of LBW piglets on D7. However, the proportions of the genes related with amino acid metabolism (7.635 vs. 8.589%, *P* < 0.05), energy metabolism (5.376 vs. 5.673%, *P* < 0.05), metabolism of cofactors and vitamins (3.560 vs. 4.023%, *P* < 0.01), biosynthesis of other secondary metabolites (0.704 vs. 0.861%, *P* < 0.05), endocrine system (0.222 vs. 0.284%, *P* < 0.05), and immune system (0.042 vs. 0.066%, *P* < 0.05) were significantly lower in the colonic microbiota of 7-day-old LBW piglets than those in the NBW ones. Genes related to membrane transport, as the most abundant pathway, were also decreased in the colonic community of 21-day-old LBW piglets (10.260 vs. 11.610%, *P* < 0.05).

### Differences in Production of the Bacterial Metabolites Between LBW and NBW Piglets

**Figure 6:**
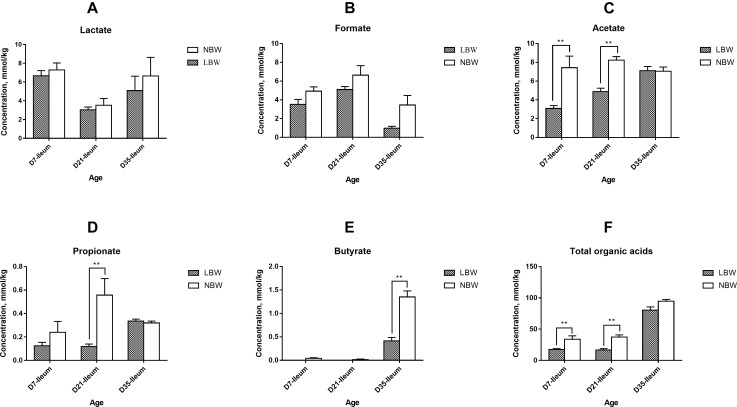
Concentrations of organic acids in ileal samples of piglets. Concentration of lactate **(A)**, formate **(B)**, acetate **(C)**, propionate **(D)**, butyrate **(E)**, and total organic acids (**F**) of LBW and NBW piglets. Data are shown as mean ± SEM. ^∗∗^*P* < 0.01. *n* = 6 per group. LBW, low birth weight; NBW, normal birth weight.

**Figure 7:**
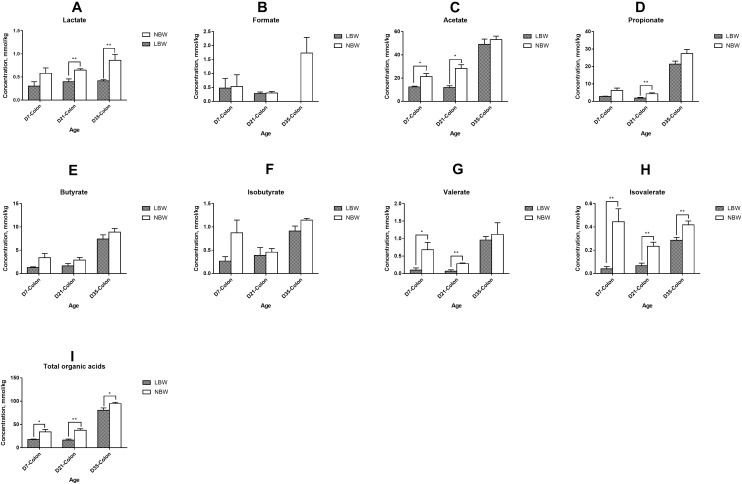
Concentrations of organic acids in colonic samples of piglets. The concentration of lactate **(A)**, formate **(B)**, acetate **(C)**, propionate **(D)**, butyrate **(E)**, isobutyrate **(F)**, valerate **(G)**, isovalerate **(H)**, and total organic acids **(I)** of LBW and NBW piglets. Data are shown as mean ± SEM. ^∗∗^*P* < 0.01; ^∗^*P* < 0.05. *n* = 6 per group. LBW, low birth weight; NBW, normal birth weight.

Total concentration of organic acids in the ileum was continuously lower than that of the colon from D7 to D35, with a significant decrease in 7- and 35-day-old LBW piglets (Supplementary Figure S4A, *P* < 0.05) and in 21- and 35-day-old NBW piglets (Supplementary Figure S4B, *P* < 0.01). In the ileum, the main organic acids consisted of lactate, formate, and acetate (Supplementary Figure S4A). The concentrations of acetate and propionate were greatly increased in the colon, while the proportion of formate was decreased (Supplementary Figure S4B). Compared with NBW piglets, LBW piglets had significantly decreased total concentration of SCFAs in the ileum from D7 and D21 (Figure 6F, *P* < 0.01) and in the colon at all three time-points (Figure 7I, *P* < 0.05). In the ileum, the concentrations of acetate on D7 and D35, propionate on D21, and butyrate on D35 were markedly reduced in LBW piglets (Figure 6C–E and Supplementary Table S5, *P* < 0.01). No difference was observed in the levels of lactate and formate between LBW and NBW piglets (Figure 6A,B and Supplementary Table S5, *P* > 0.05). In the colon, the concentrations of valerate on D7 and D21, lactate on D21 and D35, and isovalerate at each time-point were also lower in LBW piglets than those in the NBW ones (Figure 7A,G,H and Supplementary Table S5, *P* < 0.05). Additionally, the lower concentrations of acetate on D7 and D35 as well as propionate on D21 were detected again in the colon of LBW piglets compared with the normal group (Figure 7C,D and Supplementary Table S5, *P* < 0.05). No significant effect was seen in the levels of formate, butyrate, and isobutyrate, between LBW and NBW piglets (Figure 7B,E,F and Supplementary Table S5, *P* > 0.05).

### Differences in the Dominant *Lactobacillus* Species Between LBW and NBW Piglets

**Figure 8:**
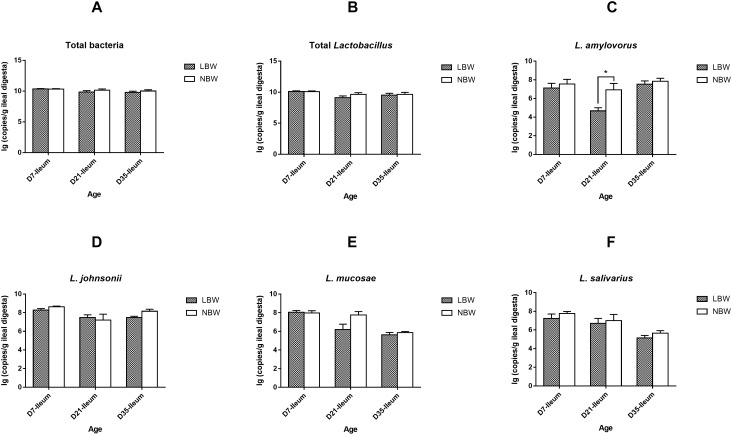
Copy numbers of predominant *Lactobacillus* species in ileal samples of piglets. The copy number of total bacteria **(A)**, total *Lactobacillus* **(B)**, *L. amylovorus* **(C)**, *L. johnsonii* **(D)**, *L. mucosae* **(E)**, and *L. salivarius* **(F)** of LBW and NBW piglets. Data are shown as mean ± SEM. ^∗^*P* < 0.05. *n* = 6 per group. LBW, low birth weight; NBW, normal birth weight.

**Figure 9:**
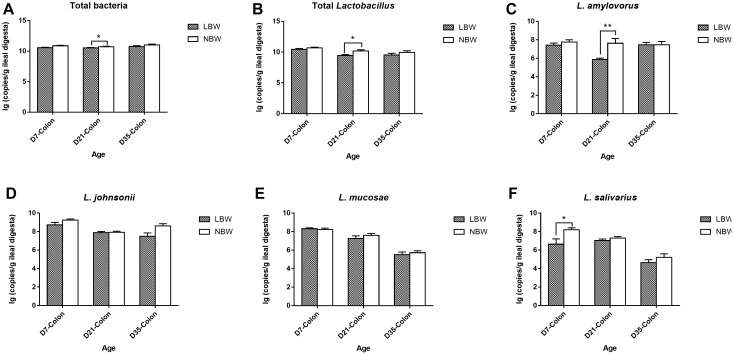
Copy numbers of predominant *Lactobacillus* species in colonic samples of piglets. The copy number of total bacteria **(A)**, total *Lactobacillus* **(B)**, *L. amylovorus* **(C)**, *L. johnsonii* **(D)**, *L. mucosae* **(E)**, and *L. salivarius* **(F)** of LBW and NBW piglets. Data are shown as mean ± SEM. ^∗∗^*P* < 0.01; ^∗^*P* < 0.05. *n* = 6 per group. LBW, low birth weight; NBW, normal birth weight.

For four predominant *Lactobacillus* species, results from qPCR analysis revealed that *L. johnsonii* had the highest copy number in both of the ileal and colonic samples, closely followed by *L. mucosae* and *L. amylovorus*, with *L. salivarius* the least (Figure 8 and Supplementary Table S6). Birth weight had no significant influence on the copy numbers of total bacteria, total *Lactobacillus*, *L. johnsonii*, *L. mucosae*, and *L. salivarius* in the ileal digesta (Figure 8A,B,D–F and Supplementary Table S6, *P* > 0.05). However, the copy number of *L. amylovorus* in the ileum of LBW piglets on D21 were significantly declined compared with the NBW ones (Figure 8C and Supplementary Table S6, *P* < 0.05). In the colon, 21-day-old LBW piglets had the less copy number of total bacteria and total *Lactobacillus* compared to NBW piglets (Figure 9A,B and Supplementary Table S6, *P* < 0.05). Moreover, the population of *L. amylovorus* was significantly reduced again in the colon of LBW piglets on D21 (Figure 9C and Supplementary Table S6, *P* < 0.01). Furthermore, the less copy number of *L. salivarius* were also observed in the colon of LBW piglets than that in the NBW ones on D7 (Figure 9F and Supplementary Table S6, *P* < 0.01). No significant influence was observed in the copy numbers of *L. johnsonii* and *L. mucosae* between LBW and NBW piglets (Figure 9D,E and Supplementary Table S6, *P* > 0.05).

## Discussion

Low-birth-weight piglets are at high risk for postnatal mortality, reduced growth rates and poor carcass quality (Wu et al., 2006; Berends et al., 2013). Our previous study has shown a significant alteration in the fecal bacterial community structure of LBW piglets during suckling and weaning periods (Li et al., 2018). Considering the segmented distribution of the gut microflora, the present investigation extended this work to the ileal and colonic bacterial community and further characterized differentially abundant taxa and microbial gene functions. In addition, we clarified spatial changes in the production of SCFAs and the colonization of predominant *Lactobacillus* species in ileum and colon between the LBW and NBW piglets. Our findings suggested that LBW piglets presented significantly altered bacterial communities, microbial metabolism and gene functions in ileum and colon from 7 to 35 days of age, especially the colonic microbiota.

Numerous studies have indicated the spatial heterogeneity in the bacterial community composition across the swine intestinal tract (Looft et al., 2014; Zhao et al., 2015; Kelly et al., 2017; Mu et al., 2017; Gao et al., 2018; Zhang et al., 2018). In our study, we found that the microbiota of ileal digesta samples from all the piglets showed lower diversity than that of colonic samples. Firmicutes and Proteobacteria were primarily colonized in the ileum, while Firmicutes and Bacteriodetes were the most prevalent phyla in the colon, which are consistent with other studies (Looft et al., 2014; Zhao et al., 2015). The spatial distribution of gut microbes may be derived from the difference in oxygen availability, pH gradient, and nutrient substrate along the intestinal lumen (Zhang et al., 2018). It has been well-known that the higher numbers of microorganisms residing in the large intestine are primarily correlated to the bacterial fermentation (DiBaise et al., 2008). In our study, microbes responsible for degrading indigestible carbohydrates, including Bacteroidetes (Arumugam et al., 2011), Prevotellaceae (Zhang et al., 2018), and some families of Firmicutes such as Lachnospiraceae (Rode et al., 1981) and Ruminococcaceae (Duncan et al., 2007), were significantly increased in the colon. The enrichment of these carbohydrate degraders, thereafter, resulted in the higher concentrations of SCFAs in the colon compared with the ileum. These observations are similar to the previous studies in piglets (Mu et al., 2017; Gao et al., 2018; Zhang et al., 2018).

Available information has shown that the gut microbiota establishment is altered in preterm infants born with LBW during early life (Fanca-Berthon et al., 2010; Arboleya et al., 2012a,b). A recent study in piglets has clarified that a significantly distinct bacterial community resides in the feces of LBW piglets during suckling and weaning periods (Li et al., 2018). Moreover, results focused on the ileum and colon have indicated greater counts of adherent bacteria in the intestinal mucosa of 2- to 5-day-old piglets born with LBW (D’Inca et al., 2010, 2011). In the current study, our findings further demonstrated that LBW significantly affected the bacterial composition in ileum and colon of the piglets from D7 to D35, especially in the colon. This suggests that selectively intervening the microflora of the hindgut may be an effective therapy to restore the altered gut microbiota of LBW piglets. There are a number of specific bacterial taxa declined in the gut of LBW piglets in this study. Compared to NBW piglets, the LBW piglets harbored lower relative abundances of Prevotellaceae, Ruminococcaceae, and Bacteroidaceae in the ileum as well as Lachnospiraceae in the colon on D7. Additionally, we observed that the proportions of *Alistipes* within Rikenellaceae, *Lachnospiraceae* FCS020 group, *Lachnostridium*, and *Eubacterium oxidoreducens* group within Lachnospiraceae, as well as *Ruminiclostridium* 9 within Ruminococcaceae, were also decreased in the colon of LBW piglets. Bacteria within these families were considered to own great abilities in degrading refractory carbohydrates to produce SCFAs (Zhang et al., 2018). *Blautia* within Lachnospiraceae and *Eubacterium nodatum* group in Clostridales are also recognized as SCFAs producers (Levine et al., 2013; Louis et al., 2014), which were less abundant in the colon of 21-day-old LBW piglets. *Desulfovibrio* spp. are generally considered as sulfate-reducing bacteria with the potential to utilize the sulfated mucins (Earley et al., 2015) and have a positive correlation with dietary fiber degradation (Luo et al., 2018). In the current study, the genus *Desulfovibrio* was discovered to have the lower prevalence in the colonic digesta of 35-day-old piglets. Therefore, the observed reduction in members of these taxa might lead to the lower production of SCFAs in the hindgut of LBW piglets. As expected, for the first time, we found that LBW piglets had lower levels of total SCFAs, acetate, propionate, valerate, and isovalerate in the colonic content at different ages compared with the normal ones. Collectively, these findings reflect an attenuated capacity to metabolize dietary fiber in LBW piglets. Besides, LBW piglets have been well recognized as being more susceptible to impaired intestinal development and various gut infections (Li et al., 2017; Wang et al., 2017). SCFAs have broad impacts on improving intestinal barrier function and reducing inflammation in the gut (Turroni et al., 2018). The reduction of SCFAs production, therefore, might partly explain the high morbidity of LBW piglets. Also, the genus *Howardella* presented a reduced relative abundance in the colon of LBW piglets at 35 days of age in our study, which is in agreement with the observation in the feces of LBW piglets (Li et al., 2018). The family Erysipelotrichaceae, which has recently been positively associated with higher feed intake (Buzoianu et al., 2012), had a decreased proportion in the colon of 35-day-old LBW piglets.

Compared to NBW piglets, the LBW piglets had higher relative abundances of *Peptostreptococcus* and *Terrisporobacter* within the family Peptostreptococcaceae in the colon on D21 and D35, respectively. Previous studies reported that Peptostreptococcaceae were more prevalent in subjects with colorectal cancer compared to controls (Chen et al., 2012; Zhu et al., 2014). This suggests that increased microbes belonging to Peptostreptococcaceae as commensal bacteria may have the potential to cause the intestinal infections of the host. Another interesting observation in the present study is that *Campylobacter* spp. of the phylum Proteobacteria, some of which are associated with the occurrence of diarrhea in piglets, were less abundant in the colonic digesta of 35-day-old LBW piglets than those in NBW piglets. However, an opposite observation was found in the feces of LBW piglets during nursing period (Li et al., 2018). These contradictory findings might result from the difference in intestinal regions and time-points sampled. *Campylobacter* spp. detected in the current study might not be pathogenic as diarrhea was not observed in any piglet born with either LBW or NBW.

Species from *Lactobacillus*, exhibiting excellent probiotic properties in improving health and disease resistance, are widely used as probiotics (Naito et al., 2011; Wang et al., 2012). Results from qPCR in this study showed that the LBW piglets had a decreased copy number of total *Lactobacillus* in the colon on D21, in agreement with previous studies in placenta, vagina, and feces of premature LBW infants (Sakata et al., 1985; Hillier et al., 1995; Zheng et al., 2015). Similar results were also seen in the feces of LBW rodents and piglets (Wang et al., 2016; Li et al., 2018). In contrast, sequencing data in this study presented a higher relative abundance of the genus *Lactobacillus* colonized in the colon of 7-day-old LBW piglets. These inconsistent results could be explained by the difference in analytical tools and targeting primers for quantification and need further investigation. Moreover, *Lactobacillus* spp. can produce lactate as a major microbial metabolite, which confers beneficial effects to the host such as inhibiting pathogen adhesion (Makras et al., 2006). In the present study, the declined concentration of lactic acid in the colonic digesta of 21-day-old LBW piglets might be partly attributed to the decrease in lactate-producing bacteria. *L. amylovorus*, widely considered as the predominant endogenous species in the gut of pigs (Pieper et al., 2006), exhibited a lower number in ileum and colon of the 21-day-old LBW piglets than that in the NBW ones. Another *Lactobacillus* species, *L. salivarius*, was also reduced in the colon of 7-day-old LBW piglets. Strains within *L. amylovorus* and *L. salivarius* as dietary probiotics can protect against infections by competitive exclusion against pathogens through bacteriocins excretion and inflammatory cytokine modulation (Messaoudi et al., 2013; Finamore et al., 2014). On basis of the above results, *Lactobacillus* spp., especially the dominant species *L. amylovorus* and *L. salivarius*, can serve as promising probiotic candidates for improving the health and growth of LBW offspring.

Beyond alterations in the microbial composition of LBW piglets, we found that the microbial gene functions of LBW piglets also differed from those of NBW piglets. Differential functional pathways between LBW and NBW piglets were mainly presented in the colonic microbiota compared with the ileum, which was consistent with the observations in the bacterial composition. Our data revealed that the functional alterations of the colonic bacterial community in LBW piglets were characterized by significantly decreased abundances of functions associated with amino acid metabolism, energy metabolism, metabolism of cofactors and vitamins, and biosynthesis of secondary metabolites on D7. Accumulating evidence indicates that the gut microbiota plays pivotal roles in amino acid catabolism and energy harvest from the diet for the generation of various bacterial metabolites including ammonia, SCFAs, and biogenic amines (Cani and Delzenne, 2009; Tremaroli and Backhed, 2012; Neis et al., 2015). Moreover, a vast array of microorganisms in the gut, such as *Lactobacilli* (Kleerebezem and Vaughan, 2009), can also act as important suppliers of various vitamins (LeBlanc et al., 2013). Furthermore, Zhang et al. (2018) have reported that these microbial pathways were positively associated with bacteria within Bacteroidetes, Lachnospiraceae, and Ruminococcaceae. Therefore, reduced proportions of these microbial pathways may reflect an impaired microbiota-mediated metabolic and biosynthetic capacity of nutrients in the intestine of LBW piglets. Nevertheless, we observed that microbial genes associated with lipid metabolism were dramatically enriched in the ileal bacterial community of 21-day-old LBW piglets. It has been evidenced that LBW newborns have a higher risk for developing adult metabolic and cardiovascular diseases due to the abnormality of fat storage and lipid metabolism (Wu et al., 2006). Therefore, the observed alteration in microbiota-associated lipid metabolism may be an important factor in the development of the metabolic disorders in later life of the LBW neonates. Moreover, genes functions involved in replication and repair, translation, poorly characterized, nucleotide metabolism, and genetic information processing were overrepresented in the colonic microbiome of 7-day-old LBW piglets compared to the NBW ones, which might cause aberrant genetic information transmission and expression in LBW piglets. Overall, perturbations in functional profiles of the gut microbiota of LBW piglets might have a long-term side effect on their physiology and health.

## Conclusion

In summary, the results of this study provide novel evidence for an alteration of the microbiome in ileum and colon of the LBW piglets. Compared with their normal littermates, LBW piglets had significantly different bacterial communities, microbial metabolism, and microbial gene functions in the ileum and colon from 7 to 35 days of age, especially in the colon. Relative abundances of some SCFAs-producing microbes, which belong to the families Bacteroidaceae, Ruminococcaceae, Prevotellaceae, and Lachnospiraceae, were dramatically decreased in LBW piglets. Reduction of these bacteria led to decreased production of SCFAs, thereby reflecting a poorer ability to ferment dietary fiber in the hindgut of LBW piglets than that of NBW piglets. Moreover, decreased numbers of *L. amylovorus* and *L. salivarius* in the gut of LBW piglets implies that these two *Lactobacillus* species could be used as potential probiotics to improve the growth and development of LBW piglets. Moreover, a clear alteration in gut microbial functionality of the LBW piglets was characterized by the altered proportions of microbial genes involved in multiple pathways such as amino acid metabolism, energy metabolism, replication and repair, and metabolism of cofactors and vitamins. This work will provide new directions in identifying the reliable biomarkers affecting early colonization of gut microbiota in LBW piglets and facilitate the development of new nutritional interventions.

## Data Availability

The datasets generated for this study can be found in NCBI Sequence Read Archive (SRA) database, SRP181998.

## Ethics Statement

This study was carried out in accordance with the recommendations of the guidelines for the Institutional Animal Care and Use Committee of China Agricultural University. The protocol was approved by the Institutional Animal Ethical Committee of China Agricultural University (CAU20170114-1, Beijing, China).

## Author Contributions

JW, ZD, and NL designed the experiments. NL, SH, and DH conducted the experiments. NL, SH, and LJ collected the samples. NL and SH performed the analysis of samples. NL, TL, and ZD analyzed the data. NL and JW wrote and revised the manuscript. All authors read and approved the final manuscript.

## Conflict of Interest Statement

The authors declare that the research was conducted in the absence of any commercial or financial relationships that could be construed as a potential conflict of interest.

## Footnotes

1 http://rdp.cme.msu.edu/

2 http://www.arb-silva.de

3 http://www.mothur.org

## Abbreviations

ANOSIM: the analysis of similarities
FDR: false discovery rate
GIT: gastrointestinal tract
IUGR: intrauterine growth restriction
LBW: low birth weight
LDA: linear discriminant analysis
LEfSe: linear discriminant analysis effect size
NBW: normal birth weight
OTUs: operational taxonomic units
PCoA: principal coordinates analysis
SCFAs: short-chain fatty acids
SEM: standard error of the mean.
