# Supplementary material for: Characterization of the Early Life Microbiota Development and Predominant Lactobacillus Species at Distinct Gut Segments of Low- and Normal-Birth-Weight Piglets
Source: Front Microbiol. 2019 Apr 16;10:797. doi: 10.3389/fmicb.2019.00797 (PMC6476964; doi:10.3389/fmicb.2019.00797)
Supplement: Supplementary file 7 [file Presentation_1.PPT]

## Slide 1
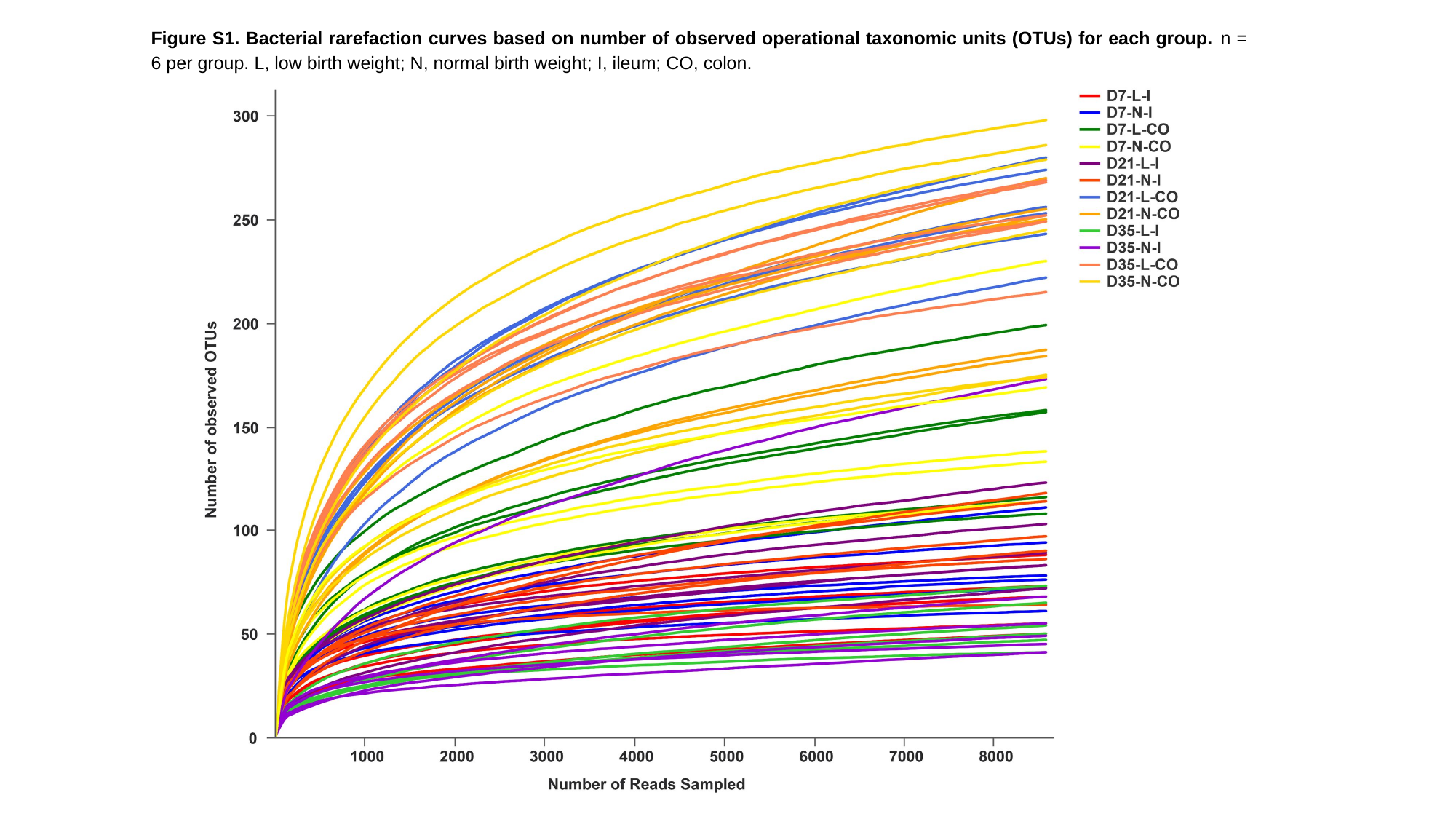

Figure S1. Bacterial rarefaction curves based on number of observed operational taxonomic units (OTUs) for each group. n = 6 per group. L, low birth weight; N, normal birth weight; I, ileum; CO, colon.

## Slide 2
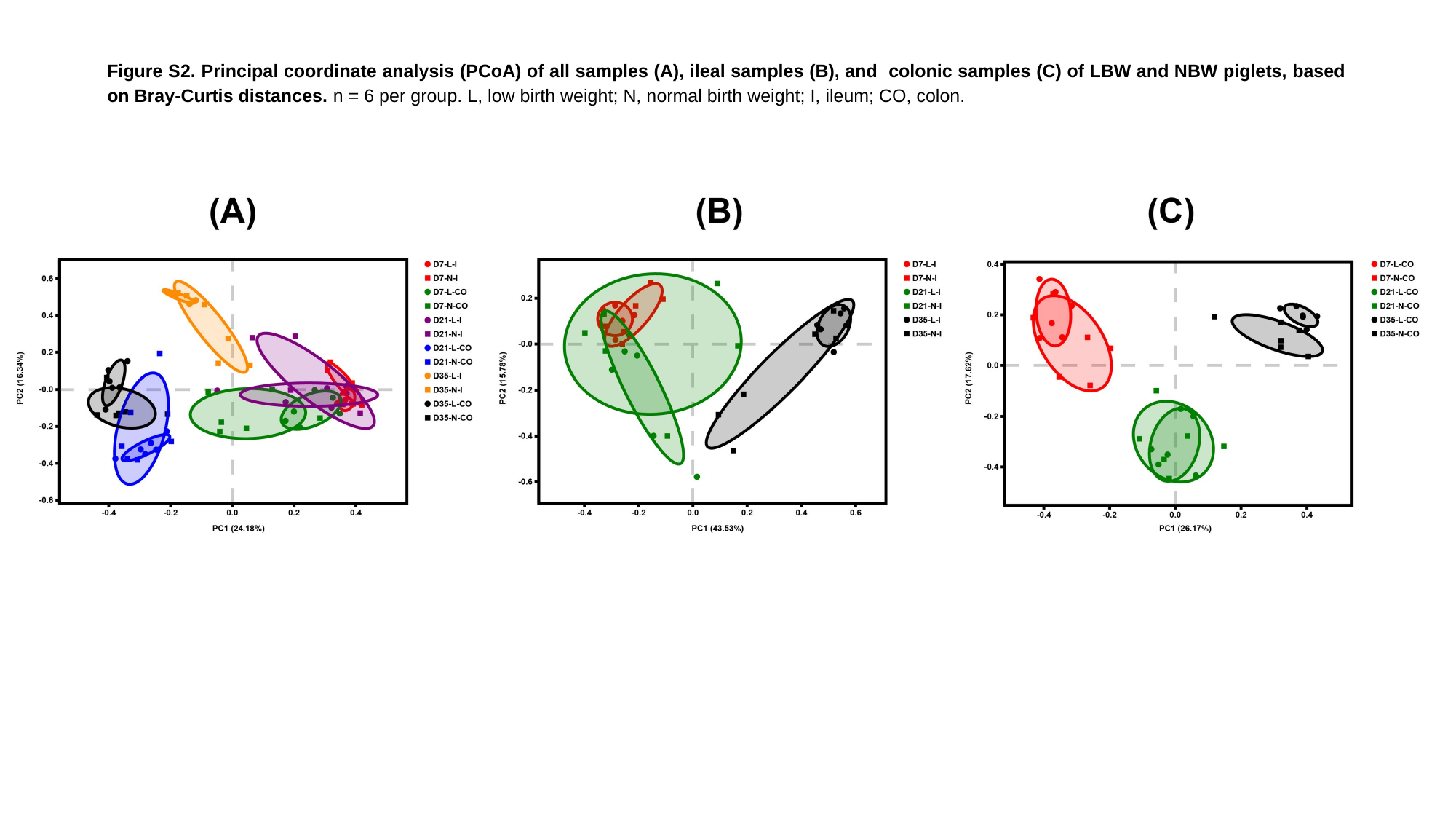

Figure S2. Principal coordinate analysis (PCoA) of all samples (A), ileal samples (B), and colonic samples (C) of LBW and NBW piglets, based on Bray-Curtis distances. n = 6 per group. L, low birth weight; N, normal birth weight; I, ileum; CO, colon.

## Slide 3
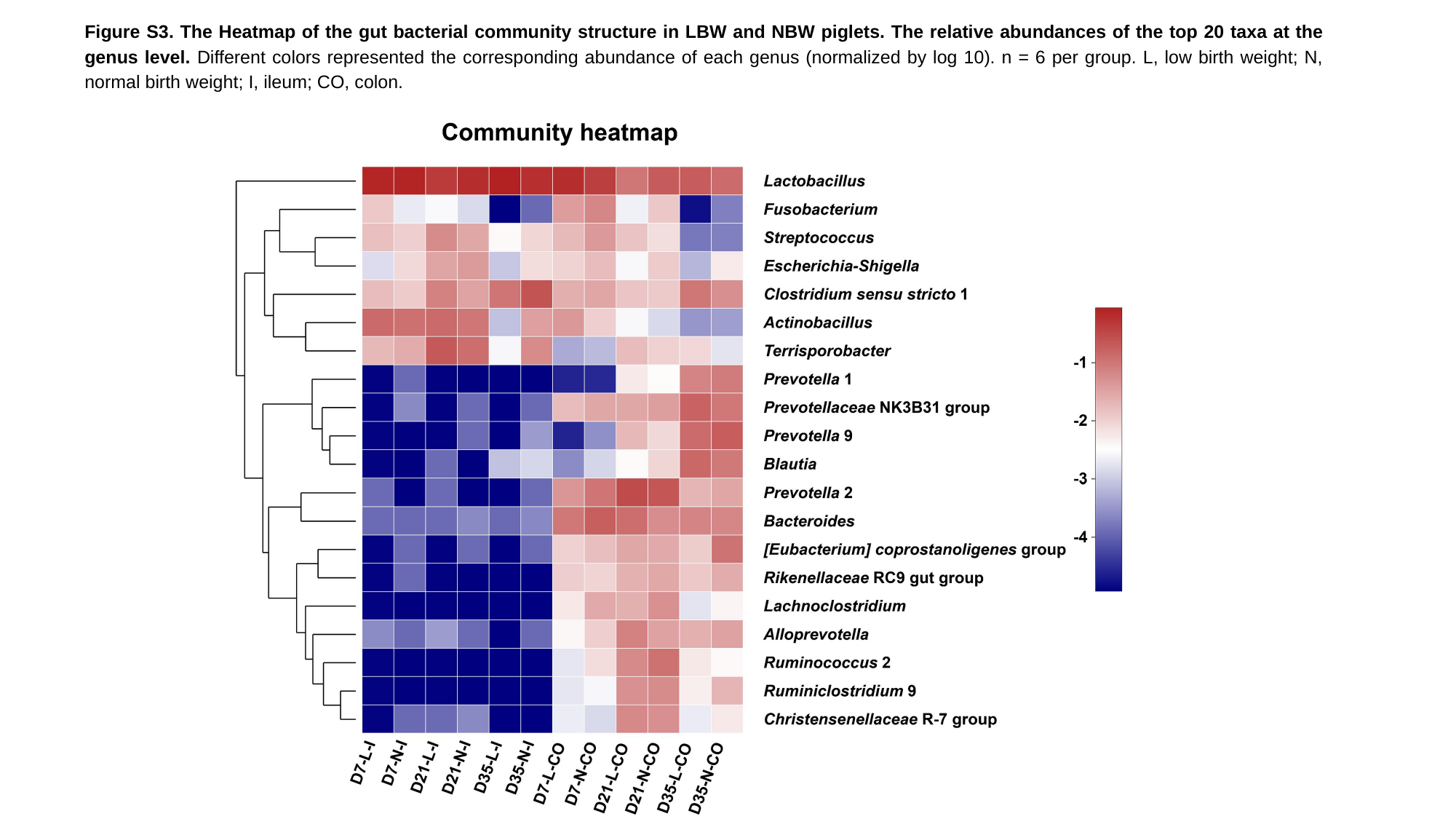

Figure S3. The Heatmap of the gut bacterial community structure in LBW and NBW piglets. The relative abundances of the top 20 taxa at the genus level. Different colors represented the corresponding abundance of each genus (normalized by log 10). n = 6 per group. L, low birth weight; N, normal birth weight; I, ileum; CO, colon.

## Slide 4
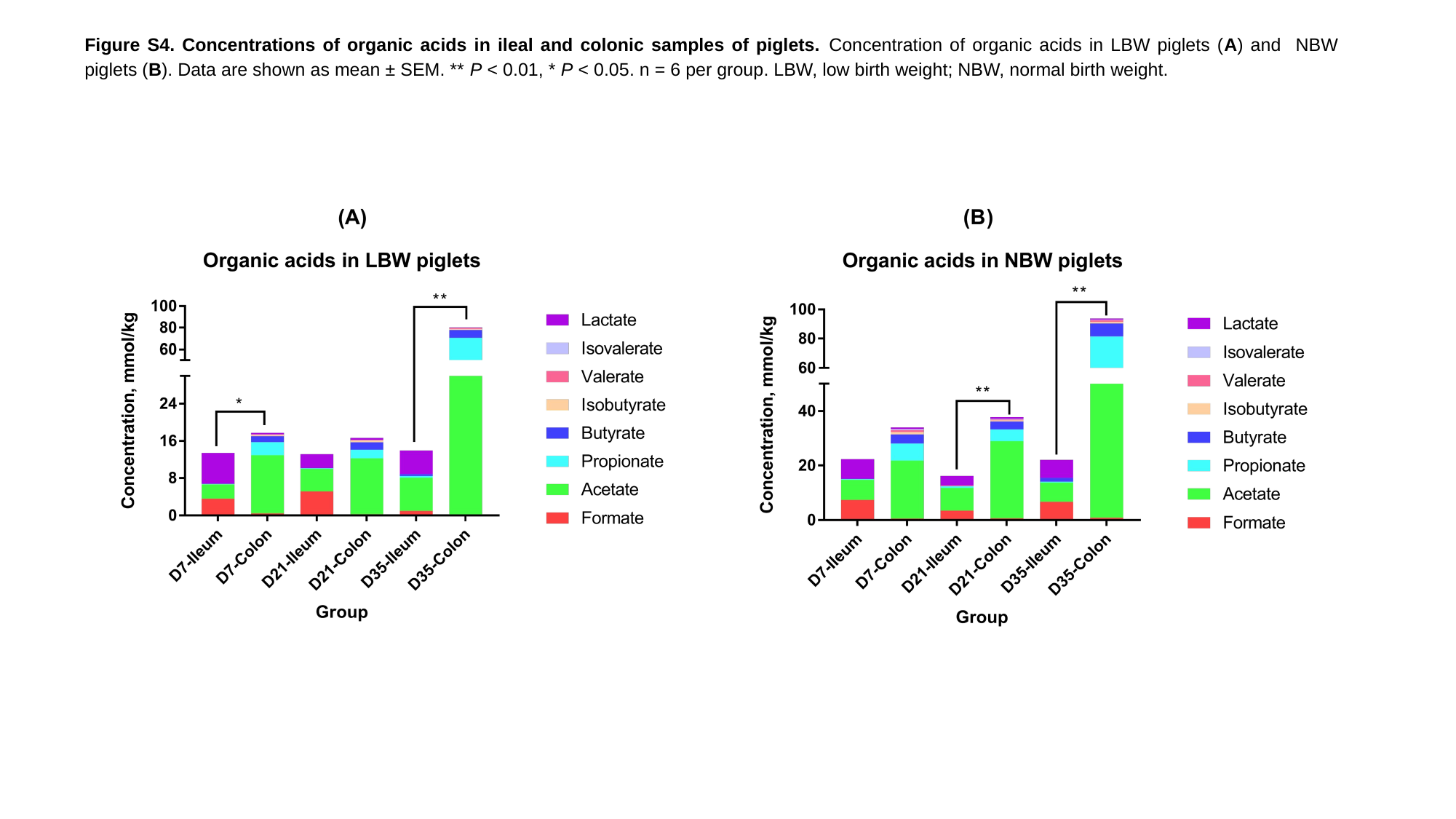

Figure S4. Concentrations of organic acids in ileal and colonic samples of piglets. Concentration of organic acids in LBW piglets (A) and NBW piglets (B). Data are shown as mean ± SEM. ** P < 0.01, * P < 0.05. n = 6 per group. LBW, low birth weight; NBW, normal birth weight.
